# Contemporary Issues in Cancer Imaging: Lung Cancer

**DOI:** 10.1038/sj.bjc.6604173

**Published:** 2008-02-19

**Authors:** N Navani, S M Janes

**Affiliations:** 1 Centre for Respiratory Research, University College of London, London, UK

Imaging has a key role in the multidisciplinary management of lung cancer, which remains the leading cause of cancer mortality worldwide. Rapid advances in imaging techniques for the diagnosis and staging of lung cancer make this book timely, not just for radiologists but also for other healthcare workers managing patients with lung cancer.

This relatively short hardback book, one of the Contemporary Issues in Cancer Imaging series, provides a tour of all the aspects of lung cancer from presentation, diagnosis and staging, to management. It begins with the clinical features followed by a chapter on the pathological differentiation of lung cancer. Histological methods of subtyping are described, including the use of immunohistochemistry. This second chapter also has 16 pages of coloured photographs that are referred to during the course of the book. The main role of imaging in the multidisciplinary meeting is for the diagnosis and staging of disease, which determine the treatment options. Non-invasive and invasive methods for staging are discussed, including the relatively novel techniques of endoscopic and endobronchial ultrasound. The advent of multi-detector row CT, accurate volumetric analysis and in particular positron emission tomography (PET) represent significant advances in the way patients are managed. A chapter on the clinical aspects of PET scanning demonstrates its role in the staging of non-small-cell lung cancer, planning of radiotherapy and evaluation of recurrence, as well as highlighting some of the issues that PET scans can raise. The book covers a wide range of basics and adds up-to-date description of the latest techniques and their uses. The nature of the topic, however, means that the field is rapidly advancing and unfortunately discussion of the most recent papers on CT screening is already notable in its absence.

The chapter dedicated to screening for lung cancer is however well balanced. The problems of over-diagnosis bias, lead-time bias and the management of indeterminate nodules are discussed. The remainder of the book contains excellent reviews on the management of lung cancer. The chapters on radiotherapy and chemotherapy provide evidence-based information that the non-oncologically trained members of the multidisciplinary team would find particularly valuable. The final chapter on surgical management details patient ‘resectability’ according to disease stage, as well as the use of neoadjuvant chemotherapy, but does not include information on the complex area of patient operability according to their physiological status.

The authors of all nine chapters are experts in their field and the text is referenced with articles up to 2005. Although most chapters begin with a conventional introduction (overlap, which may have been avoided with a specific chapter on epidemiology), the book is well written and the content easy to assimilate. Each chapter is clearly divided by sub-headings, allowing the reader to rapidly navigate to an area of interest. The key references provided are an excellent source for more detailed reading.

It is not within the scope of this book to provide a comprehensive review of lung cancer. It successfully provides a concise overview that radiologists, clinicians, pathologists, trainees and other healthcare professionals involved in the multidisciplinary management of lung cancer should find useful.

